# Defining the Proteome of Sexually Committed Parasites in *Plasmodium falciparum*

**DOI:** 10.1016/j.mcpro.2025.101505

**Published:** 2025-12-31

**Authors:** Kannan Venugopal, Fiona Achcar, Witold E. Wolski, Paolo Nanni, Leandro Lemgruber Soares, Gavin J. Wright, Matthias Marti

**Affiliations:** 1 School of Infection and Immunity, University of Glasgow, Glasgow, UK; 2 Institute of Parasitology, VetSuisse and Medical Faculties, University of Zurich, Zurich, Switzerland; 3 Functional Genomics Center Zurich (FGCZ), University of Zurich and ETH Zurich, Zurich, Switzerland; 4 Swiss Institute of Bioinformatics (SIB), Quartier Sorge - Batiment Amphipole, Lausanne, Switzerland; 5 Department of Biology, Hull York Medical School, York Biomedical Research Institute, University of York, York, UK

**Keywords:** malaria, transmission, *Plasmodium falciparum*, sexual commitment, cell separation, FACS

## Abstract

Malaria transmission from humans to mosquitoes is essential to the parasite life cycle. In the human malaria parasite, *Plasmodium falciparum*, the rate of commitment to produce the sexual transmission stages, or gametocytes varies and is governed by genetic, epigenetic and environmental factors. The sexually committed parasite has so far remained elusive due to the lack of markers to efficiently isolate these parasites for subsequent functional studies including proteomic analysis of the isolated population. Here, we demonstrate that MSRP1 is a highly specific sexual commitment marker. Using this marker, we generated and validated reporter parasite lines for subsequent FACS-based isolation of sexually and asexually committed parasites. Proteomics of isolated parasites defined distinct protein signatures, including several merozoite surface proteins, indicating functional differences between the two parasite populations. This study provides a blueprint for systematic characterization of the parasite stage at this crucial juncture in the life cycle.

## Highlights

• Generation of transgenic malaria parasites for FACS of sexually committed schizonts.
• First proteomic characterization of sexually committed schizonts.
• Differential localization validates candidate hit MSP11.
• Blueprint for systematic characterization of sexually committed schizonts.

## Graphical Abstract

**Figure:**
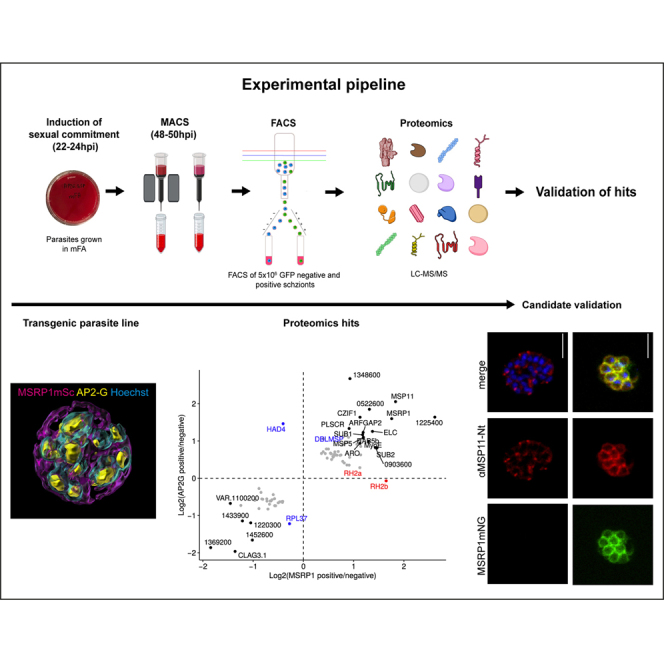

## In Brief

Malaria transmission from humans to mosquitoes is essential to the parasite life cycle yet major knowledge gaps remain. In particular the switch from asexual replication within red blood cells to formation of sexual transmission stages, i.e. sexual commitment, remains poorly understood. Here we use a transgenic reporter parasite line to isolate sexually committed cells by fluorescence-activated cell sorting for the first mass spectrometry analysis of this elusive parasite stage.

Malaria remains a major global health emergency claiming more than half a million human lives annually. In 2023 there were an estimated 263 million cases and 597,000 deaths (1). Malaria is caused by apicomplexan parasites belonging to the genus *Plasmodium*. In humans, at least five species contribute to the global disease burden, with *Plasmodium falciparum* and *Plasmodium vivax* causing the most severe forms of the disease. In *P. falciparum,* invasive forms of the parasite called merozoites invade and replicate within red blood cells (RBCs) resulting in 8 to 32 progeny that eventually egress to infect new cells. A small proportion of these asexually replicating parasites commit to produce sexual progeny. Following erythrocyte invasion, the sexual parasite stages develop as gametocytes, the only transmittable parasite stage to the mosquito. In *P. falciparum*, gametocyte development takes 10 to 12 days and is segregated into five morphologically distinct stages (Stage I-V). Only the mature Stage V male and female gametocytes are competent for mosquito transmission and subsequent reproduction. Indeed, only Stage V gametocytes are present in the blood circulation.

The malaria elimination campaign has led to renewed interest in malaria transmission as a target for interventions (2, 3). This has also inspired efforts to close knowledge gaps in the biology of the transmission stage. Sexual commitment is the first step towards onward transmission to the mosquito vector. The corresponding asexual stage is the only juncture in the parasite life cycle where two alternative outcomes are possible. The sexually committed parasite is the state that deterministically results in sexual conversion (the starting point of gametocyte-specific expression absent from any replicating blood stages) upon invasion of a new red blood cell. The rate of sexual commitment is variable, as it must be finely tuned to ensure both persistence (asexual replication) and transmission (sexual reproduction) at any given time during infection (4, 5). It is a complex process that is regulated by a combination of genetic, epigenetic and environmental factors. The transcription factor, AP2-G is an essential activator of early gametocyte genes in *P. falciparum* (6) and in *Plasmodium berghei* (7). AP2-G is in an epigenetically silenced state during asexual replication of the parasite and activated in a subset of cells upon environmental stress (6, 8, 9, 10). For example, the immune metabolite and parasite nutrient lysophosphatidylcholine (LysoPC) acts as a sensor for the parasite, and high LysoPC levels repress AP2-G activation (11). Based on these findings, we have developed a protocol to induce *P. falciparum* sexual commitment *in vitro* by LysoPC depletion for synchronous and quantitative production of gametocytes (11).

The stage of the sexually committed parasite is poorly described, not least because we lack useful tools to quantify and purify this parasite population for subsequent functional and proteomic studies (9, 10, 11, 12, 13, 14). AP2-G is expressed at low protein levels and hence AP2-G reporter parasites are barely detectable by microscopy or flow cytometry. However, an early target of AP2-G, the merozoite surface protein 7 related protein 1 (MSRP1) is detectable at the transcript and protein level in a subpopulation of parasites (10, 11, 12, 13, 14, 15). Here, we demonstrate that MSRP1 is a highly specific sexual commitment marker. Generation of AP2-G and MSRP1 reporter parasite lines enabled us to efficiently isolate sexually committed parasites for subsequent mass spectrometry analysis and comparative proteomic analysis with its asexual counterpart.

## Experimental Procedures

### Experimental Design and Statistical Rationale

Two reporter lines expressing the sexual commitment markers AP2-G and MSRP1, respectively, in combination with the early gametocyte marker GEXP02 were used in the experiment. Parasites of both lines were grown in two standard conditions (either serum or the bovine extract AlbumaxII supplemented with choline chloride) compared to minimal fatty acid medium (mFA). A biological triplicate of FACS (2-way sort – GFP/mNeonGreen positive and negative) was performed for each condition and strain, resulting in a total of 24 samples. In each experiment NF54 WT parasites with no fluorescently labeled proteins were used as a control for establishing a robust gating strategy during the FACS separation of parasite populations.

The rationale for using three replicates per condition was to balance statistical power with experimental feasibility. As detailed in the Experimental procedures section, DIA-MS data were processed with DIA-NN (v1.8.2), filtered at 0.01 precursor and 0.01 protein FDR, and aggregated to protein abundances. We applied variance stabilizing normalization before statistical modeling and imputed missing values as described. Differential expression analyses were performed using the R package ROTS, unpaired tests for comparisons between AC and serum media, and paired tests for comparing AP2-G–positive versus –negative and MSRP1–positive versus –negative samples. These choices ensured robust quantification and detection of differentially expressed proteins under each experimental condition.

### Parasite Culture

*P. falciparum* parasites of the NF54 strain (16) were cultured with AB positive RBCs at 5% hematocrit (17). The RBCs were obtained from the local blood bank Blutspende Zürich. Cultures were routinely grown in complete RPMI medium (Gibco 52400025) supplemented with 100 μM hypoxanthine (c.c.pro Z-41-M), 50 μg/ml gentamycin (Sigma-Aldrich G1397), 0.5% AlbumaxII lipid rich BSA (Gibco 11021-037), and 2 mM choline chloride (Sigma-Aldrich C7017) (10, 11). The cultures were grown in 10 ml petri dishes within a modular incubator supplied with a mixed gas formulation (5%O_2_, 5%CO_2_, and 90% N_2_). The parasites were regularly synchronized using the Percoll-sorbitol synchronization method (18), yielding a synchronicity window of up to 1 hour from the time of parasite invasion. For this purpose, parasitized RBCs were isolated at the schizont stage by performing a density gradient centrifugation using 63% Percoll (GE Healthcare GE17-0891-01). The isolated schizonts were transferred to a fresh tube, washed, and resuspended with naïve RBCs in fresh media maintaining the hematocrit at 5%. The culture was gassed and shaken at 50 rpm for 1 h at 37 °C. If the freshly formed ring parasitemia was measured to be at least 1% the culture was subject to treatment with 5% D-Sorbitol (Sigma-Aldrich S1876) for 10 min at 37 °C. The cultures were then resuspended in complete medium and maintained under standard growth conditions.

### Genetic Engineering of the Malaria Parasite

**Supplemental Figure S1:**
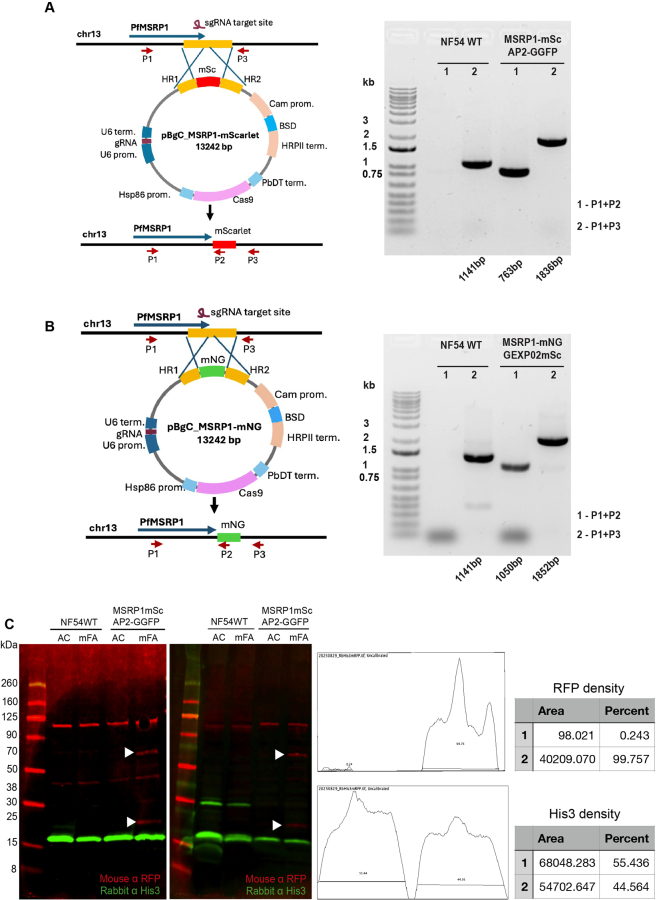


Using the single plasmid CRISPR Cas9 system (10), we constructed endogenous fluorescent tagging plasmids using the genes encoding mNeonGreen and mScarlet (pB_gC_mNeon and pB_gC_mScarlet) to tag the target genes studied here, namely *msrp1* and *gexp02*. The In-Fusion (Takara 638947) cloning method was adopted to build the plasmids (see Supplemental Fig. S1). The AP2-GGFP line previously established (11) and subsequently cloned (19) was used as the parental background in which MSRP1mScarlet and GEXP02mScarlet plasmids were transfected. In addition, a single reporter NF54_GEXP02mScarlet line (established in this study) was transfected with pD_gC_MSRP1mNeonGreen to create the NF54_MSRP1mNeonGreen-GEXP02mScarlet dual reporter parasite line. For transfections, synchronous parasite cultures with 3 to 5% parasitemia at the ring stage were harvested. Hundred micrograms of ethanol precipitated plasmid DNA was used to transfect parasites resuspended in cytomix buffer using a BioRad electroporator (20). Genetically modified parasites were selected by applying drug pressure following transfections either with 4 nM WR99210 (Jacobus pharmaceuticals, NJ) or 3 μg/ml blasticidin-S-HCl (Sigma-Aldrich 15205), dependent on the selectable marker used.

### Immunofluorescence Assays and Microscopy

Parasitized RBCs were fixed either in suspension or as thin smears on glass slides using 4% paraformaldehyde supplemented with 0.0075% glutaraldehyde (Sigma-Aldrich G5882) for 30 min at room temperature (21). Followed by a wash with PBS the cells were permeabilized with 0.1% TritonX-100 (Sigma-Aldrich) for 10 min and then washed again. The cells were then blocked with 3% BSA for 1 hour at room temperature. The blocked cells were incubated with the following primary antibodies as appropriate: chicken anti-GFP (1:1000) (Abcam 13970), rabbit anti-MSRP1 (1:500), rabbit anti-MSP11-Nt (1:1000), and R3400 rabbit anti-Rh2a/2b (1:500), diluted in blocking buffer for 1 hour at RT or overnight at 4 °C. The cells were then washed three times for 5 min each with PBS. This was followed by incubation with Alexa fluorophores (488, 594, or 647) conjugated secondary antibodies anti-chicken or anti-rabbit (Invitrogen) for a period of 1 hour at room temperature. The cells were washed once again thrice for 5 min each and then mounted under a cover slip using Vecta shield containing DAPI (Vector Labs H-1700-2). A Nikon A1R or Leica SP8 confocal microscope was used for imaging fixed samples. For super resolution microscopy, images were captured using the Zeiss Elyra SIM.

### Live Cell Imaging

Live cell imaging was performed using a Nikon A1R confocal microscope. An ibidi μ-35 mm, high Glass Bottom dish was coated with 1 ml of concanavalin-A (0.5 mg/ml, dissolved in milliQ water) (Sigma-Aldrich C2010) and incubated for 15 min at 37 °C. The concanavalin-A was then removed and the dish washed twice with PBS. NF54_AP2-GGFP-GEXP02mScarlet parasites induced for sexual commitment using mFA were grown until the culture contained predominantly mature segmented schizonts in the culture. At this point, 200 μl of culture was transferred to a 1.5 ml tube, washed once with PBS, and then resuspended in 500 μl PBS. The contents were transferred to the precoated ibidi dish and incubated for 10 min at 37 °C. Following the incubation, nonattached cells were carefully washed off and then 5 ml of complete parasite medium (pregassed) was added to the dish. The lid was quickly placed and sealed with parafilm. The dish was transferred to the imaging chamber of the NikonA1R microscope with temperature set at 37 °C. Using the Galvano scanning mode images were captured at a single plane over a time series which included captures at every 3 min for the first 30 min and then every 12 h.

### Immuno-Electron Microscopy

For immunolabeling, the samples were fixed in phosphate buffer, pH 7.2, containing 4% paraformaldehyde. After three washes in the same buffer, they were dehydrated in ascending ethanol series and embedded in LR White resin (Agar Scientific). Ultrathin sections (70 nm thick) were obtained using an ultramicrotome (Leica Microsystems). The sections were collected on formvar-coated nickel grids and then blocked in PBS containing 3% bovine serum albumin for 1 h. Afterward, they were incubated in the presence of primary antibody, washed three times in blocking buffer and incubated with 15 nm gold-conjugated Protein A (Aurion). The grids were washed three times in blocking buffer, dried, and contrasted with 4% uranyl acetate. Samples were analyzed using a JEOL 1200 EX transmission electron microscope operating at 80 kV.

### Sexual Commitment-Conversion Assays

The sexual commitment-conversion assays were performed essentially as described (22). In short, parasites were double synchronized by Percoll gradient at schizont stage and subsequently by sorbitol treatment 1 hour post invasion in ring-stage parasites. The resulting highly synchronous ring-stage parasites were continued in culture under standard growth conditions. The sexual commitment-conversion assay was set up 22 to 24 h post invasion in a 96-well plate format, with a starting parasitemia of 0.5% at 2.5% hematocrit in two separate plates. One plate was used for the sexual commitment readout, using the AP2-GGFP signal at the schizont stage. The second plate was used to quantify the parasite multiplication rate and the sexual conversion rate in the gametocyte stages. Parasites were shifted to mFA medium to induce sexual commitment and the complete medium (AlbumaxII supplemented with choline chloride) as control. The medium was changed 20 to 22 h post induction (42–46 hpi), and parasites resuspended in complete media. At this stage, the plate reserved for the sexual commitment readout was additionally treated with 50 μM E64 to block merozoite egress (23). Following 6 h of treatment with E64, the egress-arrested schizonts were stained with Hoechst 33342 and subjected to flow cytometry analysis to measure the proportion of sexually committed parasites (AP2-GGFP positive). The second 96-well plate was maintained in culture. Parasitemia was measured using Hoechst 33342 at 24 h later to calculate the parasite multiplication rate and the conversion rate using the GEXP02mScarlet signal in sexually committed ring stage parasites, by flow cytometry. At this time point, the culture media was replaced with additional supplementation of heparin to block the invasion of parasites (24) from this cycle onwards and to eliminate asexual stages. This step is essential to accurately measure the sexual conversion rate and count only gametocytes produced during the induction assay. Three days after the addition of heparin (day 5 post induction), gametocytemia was measured using the GEXP02mScarlet signal and/or the Tubulin Tracker Deep Red signal (22). Flow cytometry was performed on a BD FACS Symphony A1.

### Western Blot

Schizont stage parasites from a 10 ml synchronous culture with 2.5% hamatocrit and at 3 to 4% parasitemia were harvested by centrifugation and subject to RBC lysis by treating with 0.2% saponin. The parasite pellet was lysed using RIPA buffer (Thermo Fisher scientific) supplemented with 1X Halt protease and phosphatase inhibitor cocktail (Thermo Fisher scientific). The supernatant was separated following centrifugation at 13,000 rpm at 4 °C. Protein quantification was done using the Pierce BCA Protein Assay kit as per manufacturer’s instructions. The protein samples were prepared by mixing equal volumes of lysates with 2x Laemmli loading buffer supplemented with 2-mercaptoethanol (Bio-Rad) and boiled for 5 min at 95 °C. The samples were electrophoresed on 12% Mini-PROTEAN TGX Stain-Free protein gels and transferred onto polyvinylidene fluoride membranes using the Trans-Blot Turbo RTA Mini 0.2 μm polyvinylidene fluoride Transfer Kit as per manufacturer’s instructions (Bio-Rad). Following blocking with 1% fish gelatin in TBS with 0.1%Tween (TTBS), the membranes were probed with antibodies diluted in the same buffer. The antibodies used were rabbit anti-HSP70 (1:2000) (LubioScience LS-C129583-25), mouse anti-RFP (1:1000) (Proteintech 6G6), rabbit anti-His3 (1:2000), rabbit anti-mNeonGreen (1:1000), rabbit anti-MSP11-Nt (1:1000), and R3400 rabbit anti-Rh2a/2b (1:1000). The membranes were washed with TTBS thrice, 10 min each and then probed with IRDye fluorescent secondary antibodies (LICORbio). The membranes were once again washed in TTBS with the final wash in TBS only. The membranes were imaged on a LiCOR Odyssey F Imaging system. Quantification of band intensities for MSRP1mScarlet fusion protein and MSP11 were performed by generating densitograms using FIJI image processing tool. Normalized band intensities were calculated using the loading controls in each blot as reference, following subtraction of background noise. Western blots were done with samples harvested from a biological triplicate.

### FACS Sorting

NF54_AP2-GGFP-GEXP02mScarlet and NF54_MSRP1mNeonGreen-GEXP02mScarlet lines were used for sorting sexually committed schizonts using the BD FACS Aria S6 sorter. Following the Percoll-sorbitol synchronization method mentioned in the section sexual commitment conversion assay, parasites were induced for sexual commitment 22 to 24 hpi by shifting them to mFA medium. Parasites were cultured in a 10 ml culture volume at 2.5% hematocrit and 3 to 4% parasitemia. Forty eight to fifty hours post-induction, parasitized RBCs were enriched at the schizont stage using magnetic-activated cell sorting (MACS) columns and then subject to FACS separation. 5 × 10^6^ schizont stage parasites were sorted in a 2-way sort using an 80 μm nozzle, allowing for the collection of (AP2-G) GFP or (MSRP1) mNeonGreen positive and negative parasites. To minimize any effect on cell viability on account of exposure to the FACS sheath fluid, parasitized RBCs were directly sorted into complete medium and harvested by centrifugation. The sorted schizonts were washed two times in complete medium and then cultured under standard growth conditions to verify the ability of a AP2-G or MSRP1 positive parasites to invade naïve RBCs and convert into viable gametocytes.

### Proteomics

#### Sample Preparation

Parasites were isolated using the BD FACS Aria S6 in a 2-way sort into complete parasite medium, washed once with PBS and then lysed using RIPA buffer (Thermo Fisher 89900) (supplemented with protease and phosphatase inhibitor cocktail (Thermo Fisher 78440)). For each sample, 30 to 50 μg of denatured proteins were processed using the single-pot solid-phase enhanced sample preparation (SP3). The SP3 protein purification, digest and peptide clean-up were performed using a KingFisher Flex System (Thermo Fisher Scientific) and Carboxylate-Modified Magnetic Particles (GE Life Sciences; GE65152105050250, GE45152105050250) (25, 26). Beads were conditioned following the manufacturer’s instructions, consisting of 3 washes with water at a 1 μg/μl concentration. Samples were diluted with 100% ethanol to a final concentration of 60% ethanol. Fifty microliters of beads, the wash solutions and the samples were loaded into 96 microplates and transferred to the KingFisher instrument. Following steps were carried out on the robot: collection of beads from the last wash, protein binding to beads, washing of beads in wash solutions 1 to 3 (80% ethanol), protein digestion (overnight at 37 °C with 5 μl of trypsin (100 ng/μl in 50 mM Triethylammonium bicarbonate) and peptide elution from the magnetic beads using MilliQ water. The digest solution and water elution were combined, dried to completeness, and re-solubilized in 20 μl of MS sample buffer (3% acetonitrile, 0.1% formic acid). Peptide concentration was determined using the Lunatic UV/Vis polychromatic spectrophotometer (Unchained Labs), and all samples were normalized to an absorbance value of 0.05 using the MS sample buffer containing iRT peptides (Biognosys).

#### Liquid Chromatography Mass Spectrometry

Mass spectrometry analysis was performed on an Orbitrap Exploris 480 mass spectrometer (Thermo Fisher Scientific) equipped with a Nanospray Flex Ion Source (Thermo Fisher Scientific) and coupled to an M-Class UPLC (Waters). Solvent composition at the two channels was 0.1% formic acid for channel A and 0.1% formic acid, 99.9% acetonitrile for channel B. Column temperature was set to 50 °C. For each sample 2 μl of peptides were loaded on a commercial nanoEase MZ Symmetry C18 Trap Column (100 Å, 5 μm, 180 μm × 20 mm, Waters) followed by a nanoEase MZ C18 HSS T3 Column (100 Å, 1.8 μm, 75 μm × 250 mm, Waters). The peptides were eluted at a flow rate of 300 nl/min. After a 3 min initial hold at 5% B, a gradient from 5 to 22% B in 40 min and 22 to 32% B in additional 5 min was applied. The column was cleaned after the run by increasing to 95% B and holding 95% B for 10 min before re-establishing loading condition for another 10 min.

The mass spectrometer was operated in data-independent mode (DIA) to analyze the individual samples. DIA scans covered a range from 350 to 1050 m/z in windows of 10 m/z. The resolution of the DIA windows was set to 15,000, with a normalized AGC target value of 3,000%, the maximum injection time set to 23 ms, and a fixed normalized collision energy (NCE) of 28%. Each instrument cycle was completed by a full MS scan monitoring 350 to 1′050 m/z at a resolution of 120,000. The mass spectrometry proteomics data were handled using the local laboratory information management system B-fabric (27), and all relevant data have been deposited to the ProteomeXchange Consortium via the PRIDE (http://www.ebi.ac.uk/pride) partner repository with the data set identifier PXD059080 (AD1) (28).

#### Protein Identification and Quantification with DIANN and DEA

The acquired DIA MS data were processed for identification and quantification using DIANN v1.8.2 (29). Spectra were searched using the protein database in FASTA file format of the *P. falciparum* reference genome 3D7 (a clone of NF54), common protein contaminants, and the target proteins AP2-G and MSRP1 and their reporters (5897 protein sequences) in FASTA file format. Carbamidomethylation of cysteine was fixed, while methionine oxidation was variable. Enzyme specificity was set to trypsin/P, allowing a minimal peptide length of 7 amino acids and a maximum of two missed cleavages. The R package prolfqua (30) was used to determine protein abundance and normalize the data. We started with the “report.tsv” file, which was generated by DIA-NN and reports the precursor ion abundances for each raw file. The mass tolerance for precursor ions was 15 ppm and the mass tolerance for fragment ions was 20 ppm. We filtered at 0.01 precursor FDR and 0.01 Protein FDR. We determined protein abundances by first aggregating the precursor abundances to peptidoform abundances. Then, we employed the Tukeys-Median Polish to estimate protein abundances. Furthermore, before fitting the linear models to determine the group differences, we transformed the protein abundances using the variance stabilizing normalization (31) (Supplemental Table S4).

The missing values were imputed from the normalized abundances using the average expression at percentile 0.05 of the group where the protein is not quantified. The differential expressions were calculated using the R package ROTS (version 1.32.0) (32, 33). The four tests comparing protein expression in AC and serum media (for each type of samples: AP2G or MSRP1 positive and negative) were done using unpaired tests. The two tests comparing AP2G positive to negative and MSRP1 negative and positive were done using paired tests (pairing the samples originating from the same culture). The false discovery rates and log2 fold changes returned by ROTS were used thereafter. The volcano plots were done using the R packages ggplot2 (https://github.com/kassambara/ggpubr; https://cran.r-project.org/web/packages/ggrepel/index.html) (34).The heatmaps were done using the R package pheatmap (https://github.com/raivokolde/pheatmap).

## Results

### MSRP1 is Expressed in Sexually Committed Schizonts

**Fig. 1:**
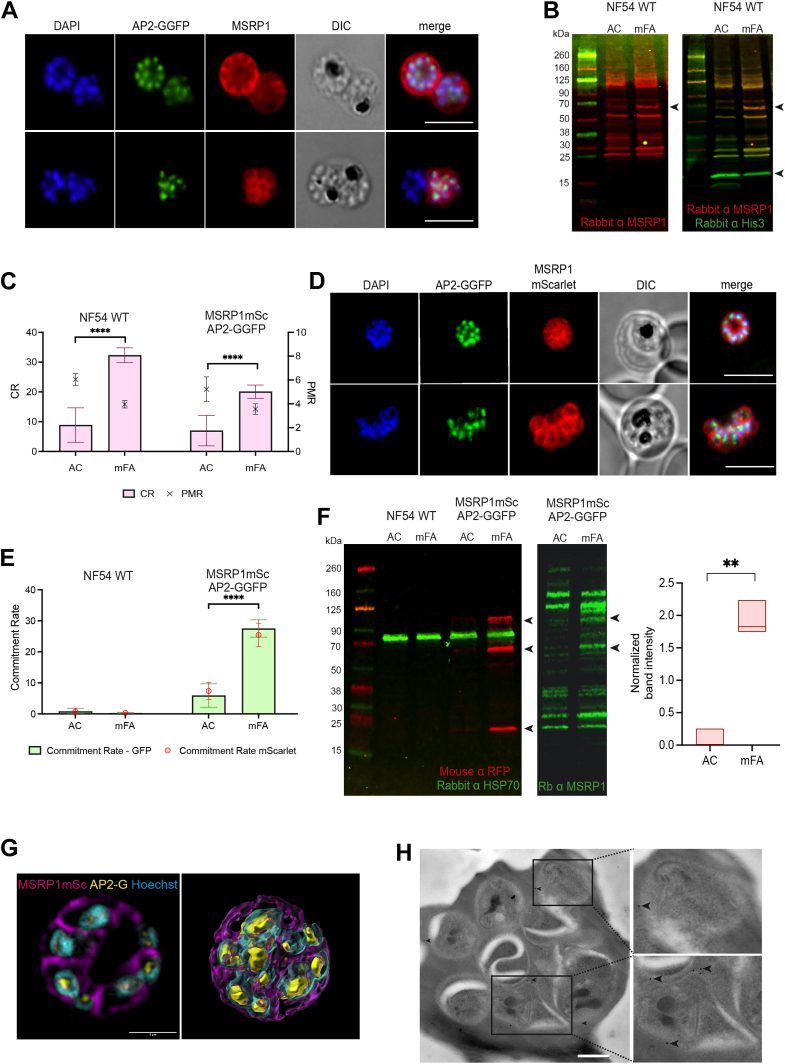
**Establishment of reporter lines for the identification of sexually committed schizonts.***A*, confocal microscopy images of MSRP1 positive schizonts upon mFA induction. Shown are representative images of AP2-GGFP (nuclear localization) costaining with MSRP1 labeled with a rabbit polyclonal antibody (peripheral localization), nuclear stain (DAPI, *blue*), and differential interference contrast (DIC) in the same cells. Scale bar represents 5 μM. *B*, Western blot analysis of MSRP1 expression on NF54 WT schizont samples of induced (mFA) and uninduced (AC) populations. Membranes were probed with anti-MSRP1 rabbit polyclonal antibody and anti-Histone H3 (His3) rabbit polyclonal antibody (loading control). *Arrowheads* mark the loading control at ∼15 kDa and an induced band for MSRP1 under mFA conditions at ∼60 to 70 kDa band. Notably, MSRP1 has a predicted size of 42.9 kDa. *C*, sexual conversion rate (CR) and parasite multiplication rate (PMR) of parental NF54 WT and dual reporter *NF54 MSRP1mSc_AP2-GGFP* when grown under uninduced (AC) or inducing conditions (mFA) using flow cytometry. CR was measured using Tubulin Tracker Deep Red and PMR was measured using the nuclear dye Hoechst. n = 3*, p*-value <0.0001 using two-way ANOVA. *D*, confocal microscopy images of MSRP1mScarlet positive schizonts upon mFA induction in the *MSRP1mSc_AP2-GGFP* dual reporter line. Shown are representative images of AP2-GGFP (nuclear localization) costaining with MSRP1mScarlet (peripheral localization), nuclear stain (DAPI, *blue*), and differential interference contrast (DIC) in the samecells. Scale bar represents 5 μM. *E*, comparison of sexual commitment rate measurement using AP2-GGFP and MSRP1mSc reporters by flow cytometry. NF54 WT parasites were used as a negative control for the fluorescent markers. Parasites were grown under uninduced (AC) and inducing conditions (mFA). Data were analyzed using two-way ANOVA. *F*, representative Western blot of the same four parasite populations as in (*E*). *Left*: Detection of MSRP1mScarlet with mouse monoclonal RFP antibody. Rabbit polyclonal anti HSP70 (running at ∼ 70 kDa) was used as a loading control. In a separate blot detection of MSRP1mScarlet with rabbit polyclonal, MSRP1 is shown. *Arrowheads* indicate the MSRP1mScarlet fusion protein (at ∼95 kDa), MSRP1 (at 60–70 kDa), and mScarlet only (at 25 kDa). *Right*: Quantification of mFA-induced MSRP1mScarlet expression in the dual reporter line in comparison with the lysates of AC treated (uninduced) parasites. Data were analyzed using unpaired *t* test with Welch’s correction. *G*, 3D-SIM super resolution microscopy of schizonts upon mFA induction. AP2-GGFP (*yellow*), Hoechst (*cyan*), MSRP1 (*magenta*). *Left* panel: unprocessed image. *Right* panel: 3D rendering by IMARIS. Scale bar represents 5 μM. *H*, immuno electron microscopy of schizonts upon mFA induction. Mouse monoclonal anti-RFP (gold particles pointed by *arrow heads*) was used to localize the MSRP1mScarlet reporter protein. Scale bar represents 500 nM. AC, AlbumaxII-choline chloride medium; mFA, minimal fatty acid medium. ∗∗∗∗*p* < 0.0001, ∗∗∗*p* < 0.001, ∗∗*p* < 0.01.

AP2-G is expressed in the sexually committed schizont and early gametocyte stages, activating target genes during both phases of the parasite cycle (6, 14). A series of recent studies have identified *msrp1* as a putative transcriptional marker of sexual commitment (11, 12, 13, 14). Indeed, *msrp1* is an AP2-G target gene in schizont stages. To determine whether MSRP1 protein is uniquely expressed in sexually committed parasites, we analyzed MSRP1 expression using a polyclonal antibody generated against recombinant MSRP1 (35) in an AP2-G reporter line, the *NF54_ap2-ggfp* line (11, 19). By fluorescence microscopy we were able to detect MSRP1 expression in a subset of parasites that were also co-stained for AP2-G (Fig. 1*A*), suggesting that it indeed labels sexually committed schizonts. To confirm the specificity of the antibody, we analyzed protein expression by Western blot (Fig. 1*B*). Probing the membrane with the MSRP1 antibody in NF54 wild type parasites revealed multiple bands, including one at ∼60 to 70 kDa that was uniquely increased under induced conditions compared to control.

We therefore generated a MSRP1 reporter line by introducing a C-terminal mScarlet reporter at the *msrp1* endogenous locus, resulting in the transgenic dual reporter parasite line *NF54_msrp1mScarlet_ap2-ggfp* (see Supplemental Table S1 for details). This dual reporter line enabled us to investigate the expression and localization of MSRP1 in live cells compared to AP2-G expression and asexual control parasites. The dual reporter line was confirmed by genotyping PCR (Supplemental Fig. S1) and individual clones were induced for stage conversion using LysoPC depletion, demonstrating that the dual reporter line has a sexual conversion rate of approximately 20% (Fig. 1*C*). Signal of the fusion protein was detected by IFA (Fig. 1*D*) and quantified by flow cytometry, demonstrating co-expression in the subset of sexually committed cells that are positive for AP2-GGFP (Fig. 1*E*). Western blot analysis using a reporter antibody (monoclonal anti-RFP mouse antibody to detect mScarlet) also revealed a major increase of signal for the fusion protein upon induction (Fig. 1*F*, Supplemental Fig. S1). Notably, the polyclonal MSRP1 antibody also detected the fusion protein by Western blot specifically in induced parasites. Next, we employed 3D Structured Illumination Microscopy (SIM) to investigate MSRP1mScarlet localization at the nanoscale. These experiments demonstrated that MSRP1 localizes at the merozoite surface, while revealing a perinuclear distribution of AP2-G (Fig. 1*G*). The localization of MSRP1 was confirmed by immunoelectron microscopy using the monoclonal anti-RFP antibody (Fig. 1*H*). Altogether, these data validate MSRP1 as a marker for sexual commitment in *P. falciparum*. In comparison with the expression of the transcription factor AP2-G, the merozoite surface protein MSRP1 is highly expressed and easily detectable by fluorescence microscopy, flow cytometry and Western blot.

### MSRP1 and AP2-G Reporter Enable Quantification of Sexual Commitment Rates

**Fig. 2:**
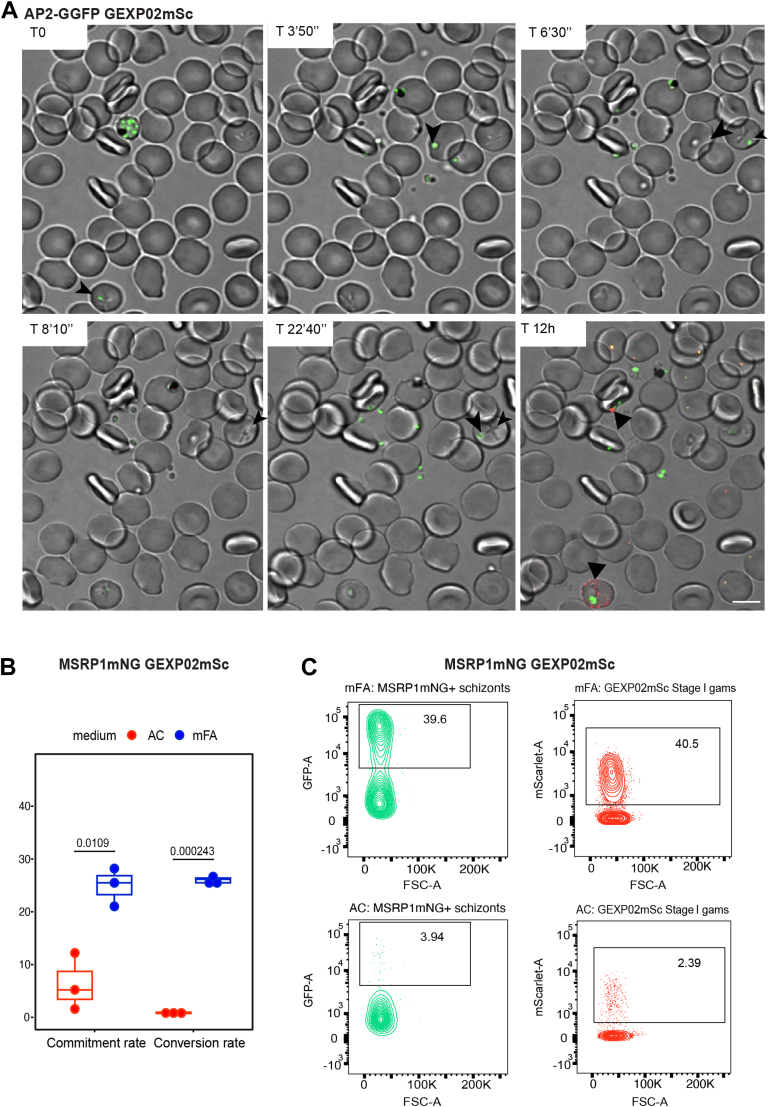
**Validation of MSRP1 and AP2-G reporter lines for fluorescence-activated cell sorting of sexually committed schizonts.***A*, time stamps (TS) from a 12-h time course of time lapse imaging using the AP2-GGFP_GEXP02mScarlet line. The experiment was started in late schizont stages upon mFA induction. Shown are green merozoites from a sexually committed schizont (AP2-GGFP positive) before egress (TS = 0) and during invasion (T 3′50” and 6′30”). Ring stage development is marked at T8′10” and 22′40”. RBC invasion of *green* parasites and activation of *red* (GEXP02mScarlet positive) gametocytes are shown in TS = 12h. Green merozoites and ensuing rings are tracked with a *black arrow* and a *red* gametocyte is marked with a *black arrowhead*. Scale bar represents 5 μM. *B*, boxplot showing commitment and conversion rate of the NF54_MSRP1mNeonGreen-GEXP02mScarlet line grown in AC and mFA media, respectively. *p*-values are based on unpaired t-tests. *C*, representative flow cytometry scatter plots for the NF54_MSRP1mNeonGreen-GEXP02mScarlet line grown in mFA medium (*top*) and AC medium (*bottom*). Gating for sexually committed schizonts based on MSRP1mNG expression (*left*) and GEXP02mSc expression in Stage I gametocytes (*right*).

We recently established a dual reporter line combining an AP2-G reporter with a reporter of gametocyte exported protein 2 (GEXP02) (36, 37, 38), creating *NF54_ap2-ggfp_gexp02mScarlet* (22). This dual reporter line allowed us to directly correlate the rates of sexual commitment and conversion. *gexp02* is an AP2-G target gene and expressed in gametocytes starting from 12 to 16 h post invasion. Indeed, time lapse imaging of the *NF54_ap2-ggfp_gexp02mScarlet* line directly demonstrated that GFP-positive parasites (*i.e.*, sexually committed schizonts) become GFP-positive again upon reinvasion (*i.e.* sexual rings) during early gametocyte development. The GFP signal then faded while the mScarlet signal from the GEXP02 reporter became brighter (Fig. 2*A* and Supplemental Movie S1).

**Supplemental Figure S2:**
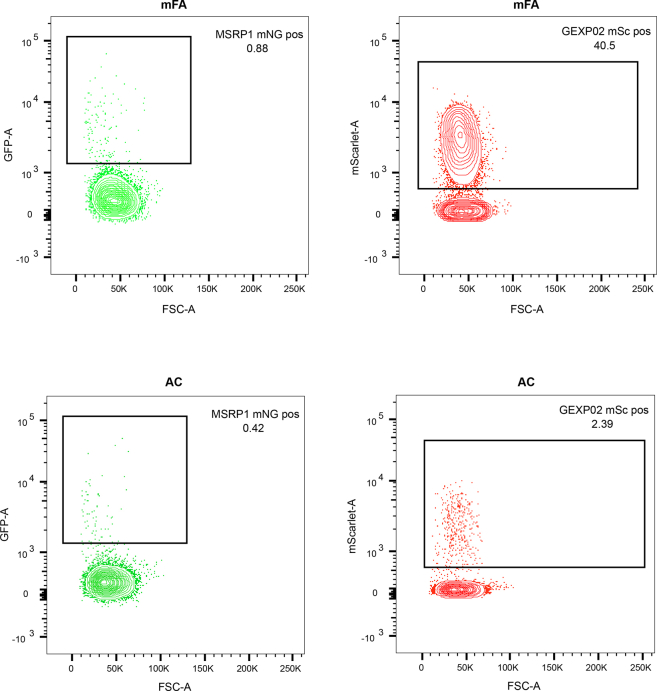


To independently validate the correlation between commitment and conversion, we generated another dual reporter line combining the MSRP1 reporter with a GEXP02 reporter, resulting in *NF54_msrp1mNeonGreen_gexp02mScarlet* (see Supplemental Fig. S1 for plasmid map). Indeed, sexual commitment and conversion rates measured by flow cytometry were identical in this line, demonstrating that both AP2-G and MSRP1 are excellent markers for sexual commitment while GEXP02 is an excellent marker of sexual conversion (Fig. 2, *B* and *C*). Notably, MSRP1 could not be detected upon reinvasion by flow cytometry analysis of ring-stage parasites (see Supplemental Fig. S2), suggesting that the protein is not expressed in early gametocytes, unlike AP2-G or GEXP02.

### Proteomic Analysis of Sexually and Asexually Committed Parasites Isolated by Fluorescence-Activated Cell Sorting

**Fig. 3:**
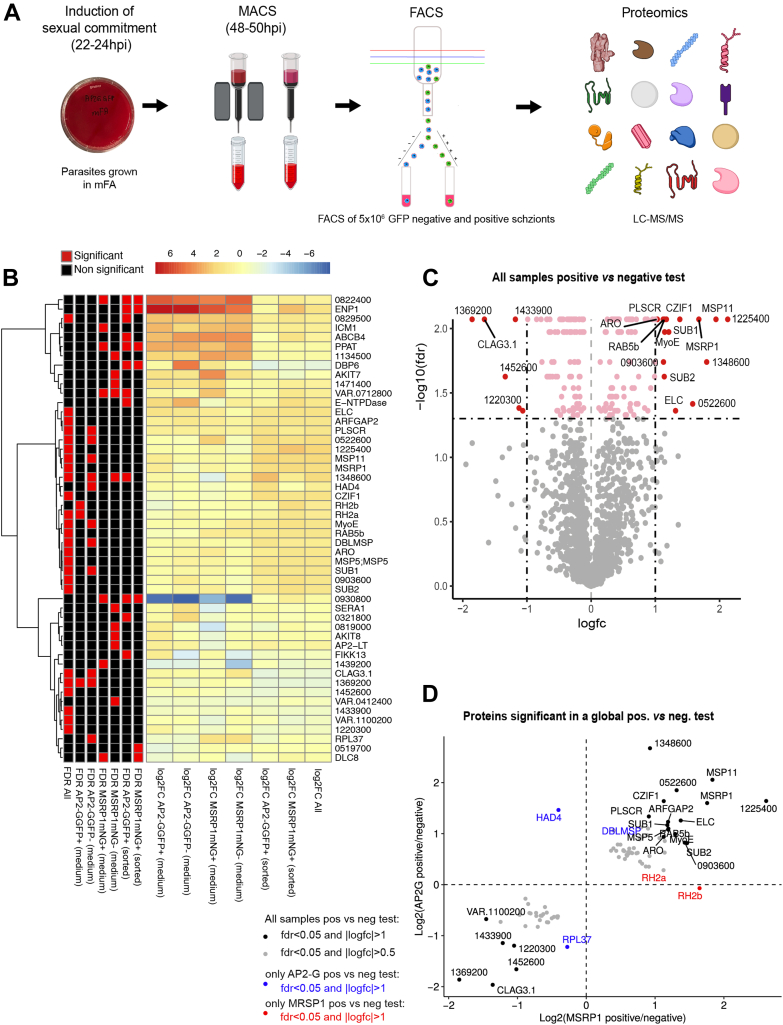
**Proteomic analysis of sexually versus asexually committed schizonts.***A*, workflow for proteomic analysis of FACS-based isolation of sexually committed and noncommitted schizonts. Parasites were induced for sexual commitment in 10 ml cultures in mFA medium at 2.5% hematocrit with 3 to 4% parasitemia. At the schizont stage, parasites were separated from uninfected RBCs by MACS and then subject to FACS-based separation. *B*, heatmap based on log2 fold change from four tests comparing medium condition per sorted parasite population (AC *vs* serum, positive log fold change = up in AC) and two tests comparing sorted populations only (positive log fold change = up in the positive samples, *i.e.*, MSRP1mNeonGreen positive or AP2-GGFP positive parasites). Included are all significant hits from at least one comparison with FDR<0.05 and |log2fc|>1, ordered using hierarchical clustering (method = ward.D, distance = euclidean). See Experimental procedures for details. *C*, volcano plot of the paired tests comparing all positive versus negative samples across the two parasite lines, using the same method as in (*B*) (see Experimental procedures). In *red* are statistically significant hits with |log2 fold change| > 1. *D*, proteins with statistically different abundancies between all positive *vs* negative samples (with FDR<0.05 and |log2fc|>0.5 for the global paired test, see Experimental procedures). x-axis is the log2 fold change of AP2-G positive *vs* negative; y-axis: log2 fold change of MSRP1 positive *vs* negative. The *blue dots* show proteins that are only significant for the AP2-G test (FDR<0.05 & |log2fc|>1), *red* are proteins only significant for the MSRP1 test, *black* are significant for both (FDR<0.05 & |log2fc|>1). Full results for data shown in (*B–D*) are available as Supplemental Table S2.

Having validated two *P. falciparum* lines that enable detection and quantification of sexually committed schizonts by flow cytometry opened the possibility of fluorescence-activated cell sorting (FACS) for further characterization of this elusive parasite stage. Therefore, we used both dual reporter lines (*i.e.*, *NF54_ap2-ggfp_gexp02mScarlet* and *NF54_msrp1mNeonGreen_gexp02mScarlet*) to establish a reproducible protocol for FACS and subsequent proteomic analysis. Synchronized parasite cultures were induced for sexual commitment in mFA as described (11) and schizont stages separated from uninfected RBCs using MACS, taking advantage of the ferromagnetic properties of hemozoin in the parasite. The enriched schizont population was then subjected to FACS to separate MSRP1mNeonGreen-positive and AP2-GGFP-positive schizonts, respectively, from their negative counterparts (Fig. 3*A*). Following parasite lysis, the lysates were subjected to liquid chromatography coupled to tandem mass spectrometry. Parasites cultured in mFA have a lower parasite multiplication compared to those grown under standard conditions due to limited nutrient supply (12). We therefore only compared sorted parasites upon induction in mFA. We performed two separate experiments in triplicate where parasites were routinely grown in two different standard media, namely incomplete RPMI-HEPES medium supplemented either with human serum or with AlbumaxII and choline-chloride. Parasites were grown in either of the two standard media prior to induction of sexual commitment by shifting the parasites to mFA medium. Running parallel proteomics experiments using these two standard media enabled us to control for potential confounders based on nutrient supply.

This analysis identified 1980 proteins with at least one peptide each as coverage in one of the two lines (Supplemental Table S2). Comparison of protein abundances between sorted populations (positive *vs* negative fluorescence; labeled as “sorted” in Fig. 3*B*) or across medium conditions (labeled as “medium” in Fig. 3*B*) identified proteins with significant differences in at least one out of these six comparisons (Fig. 3*B* and Supplemental Table S2). The largest differences were observable between medium conditions, regardless of how parasites were sorted based on fluorescence. Focusing on the differences between sorted populations, we controlled for medium condition and combined the data from the AP2-G and MSRP1 reporter lines. This analysis revealed a small set of 22 (*p* < 0.05, |log FC|>1) and 81 proteins (*p* < 0.05, |log FC|>0.5), respectively, with significantly different abundance between fluorescence-positive (sexually committed) and fluorescence-negative populations (Fig. 3*C* and Supplemental Table S3).

Of the candidate proteins, 16 (52 at *p* < 0.05, |log FC|>0.5) of them were enriched in sexually committed schizonts and 6 (29) in non-committed schizonts. The 16 candidates enriched in sexually committed schizonts included three proteins linked to erythrocyte invasion (MSP5, MSP11, and ELC) and five linked to protein trafficking (ARFGAP2, Rab5B, PLSCR, MyoE, and ARO). By contrast, several components of the DNA replication complex MCM and the PSAC (Plasmodium Surface Anion Channel) component Clag3.1 were enriched in non-committed schizonts (Fig. 3, *C* and *D* and Supplemental Table S3).

### Validation of Proteomics Hits by Fluorescence and Electron Microscopy

Several of the RBC invasion proteins significantly enriched in sexually committed schizonts are known merozoite surface proteins (MSPs). Apart from MSRP1, these include MSP5 and MSP11. At the lower cutoff we also identified DBLMSP, MSP2 and MSP6, as well as the reticulocyte binding-like homolog Rh2a, which is released from the rhoptries during RBC invasion (39, 40, 41, 42). To validate our findings from the proteomics data, we performed a series of immunofluorescence assays. Specifically, we produced sexually committed and non-committed schizont populations of the *NF54_msrp1mNeonGreen_gexp02mScarlet* line, analogous to those generated for FACS and subsequent mass spectrometry analysis. Parasites were then fixed and either probed with polyclonal antibodies raised against MSP11 (43) or Rh2a/b (binds to the conserved C terminal domain) (42).

**Fig. 4:**
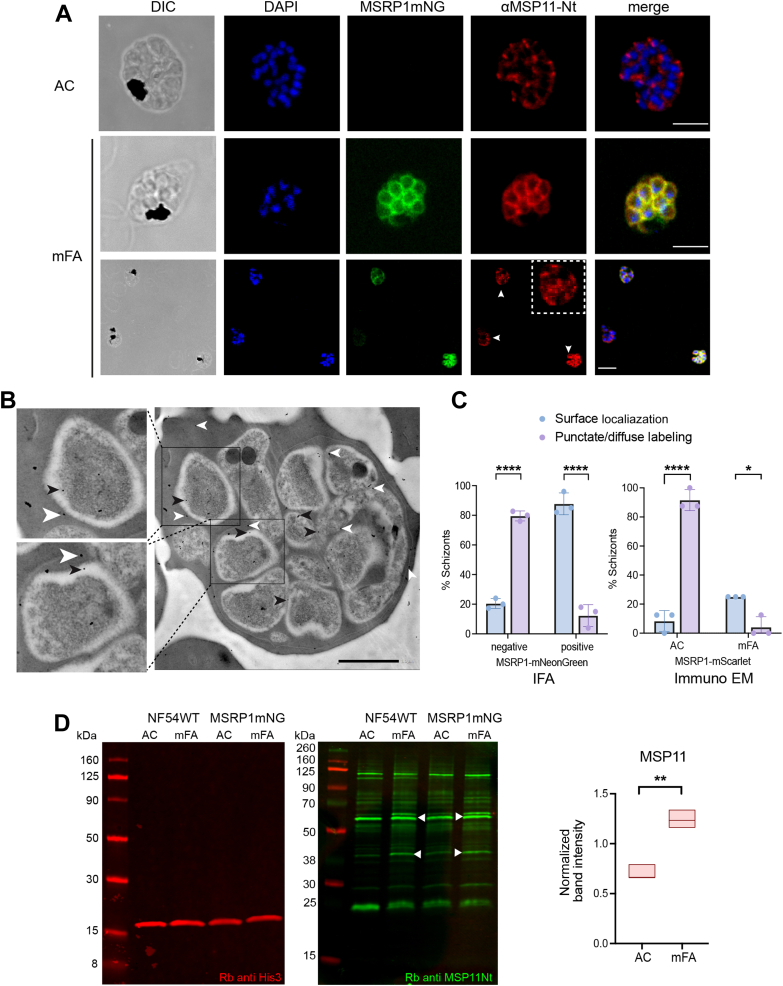
**Validation of candidate markers for sexually and asexually committed schizonts.***A*, confocal microscopy image panels of MSRP1mNeonGreen parasites costained with candidate markers. Parasites were grown in AC or mFA media, respectively, and costained with MSP11 antibodies. Representative images of differential MSP11 distribution in AC (apical punctate pattern) *vs* mFA (surface colocalization with MSRP1) are shown in the *top panel*. The third row shows three parasites with three staining patterns: punctate staining in an MSRP1 negative schizont, diffuse staining in a schizont with weak MSRP1 signal and merozoite surface localization in MSRP1-positive schizonts. Scale bar represents 5 μM. *B*, immuno electron microscopy image of a schizont upon mFA induction where MSP11 localization is labeled with 25 nm gold particles coated with MSP11Nt antibody (*white arrowheads*) and MSRP1 localization is labeled with 15 nm gold particles coated with RFP monoclonal antibody (*black arrowheads*). Two merozoites are enlarged to demonstrate the surface localization of the gold particles. Scale bar represents 1 μM. *C*, quantification of MSP11 localization. *Left panel*: Localization by IFA in FACS sorted parasites upon induction. The surface and punctate/diffuse pattern of localization was quantified in both MSRP1-mNG positive and negative parasites. Data were analyzed using R function prop.test. *Right* panel: Localization by immuno EM. The surface and punctate/diffuse pattern of localization was quantified in AC and mFA-treated parasites. Data were analyzed using the raw cell counts and fdr calculated using R function pairwise.prop.test. *D*, Western blots probed with Histone H3 (His3) antibody as loading control followed by probing of MSP11-Nt antibody. The 40 kDa band corresponding to MSP11 and an additional specific band identified previously at app. 60 kDa (43) using the rabbit anti MSP11-Nt antibody (*green*) are indicated by *arrowheads*. Quantification using normalized band intensity from a biological triplicate is shown. Data were analyzed using unpaired *t* test with Welch’s correction. ∗∗∗∗*p* < 0.0001, ∗∗∗*p* < 0.001, ∗∗*p* < 0.01, ∗*p* < 0.1.

**Supplemental Figure S3:**
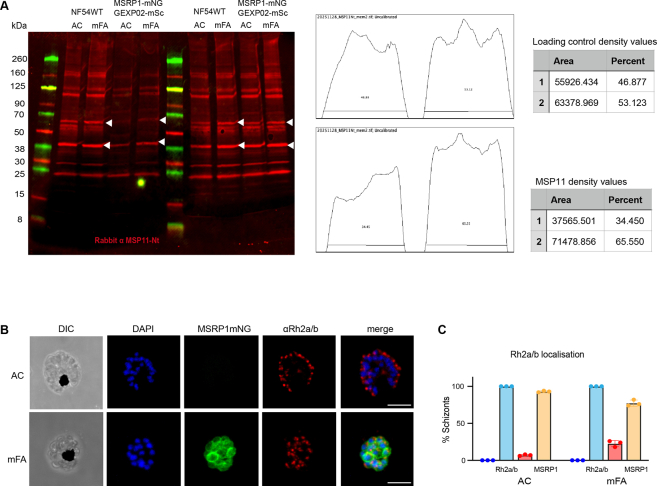


Using MSP11 antibodies, we observed a differential staining pattern between sexually committed and non-committed schizonts. In sexually committed schizonts, the protein consistently colocalized with MSRP1 on the merozoite surface. In non-committed schizonts, however, it showed a punctate localization at the apical tip of the merozoite (Fig. 4*A*). A third diffuse MSP11 localization was observed in a few schizonts weakly stained with MSRP1, possibly representing MSP11 protein *en route* to the merozoite surface. We further validated this observation by performing immuno EM experiments on MSRP1mScarlet parasites in standard and induced conditions (Fig. 4*B*). Importantly, quantification of IFA signal distribution from FACS sorted parasites and of gold particles by immuno EM confirmed that the majority of MSRP1 positive schizonts showed MSP11 surface localization, while those without MSRP1 staining predominantly showed punctate or diffuse internal staining (Fig. 4*C*). These data therefore demonstrate differential localization of MSP11 in sexually committed *vs* non-committed schizonts. Quantitative analysis of protein expression by Western blot revealed a significant increase in MSP11 expression under inducing conditions both in WT and MSRP1 reporter parasites, confirming the proteomics data (Fig. 4*D*). By contrast, we did not observe any difference in localization or staining intensity when using the Rh2a/b antibody in sexually committed *versus* non-committed schizonts (Supplemental Fig. S3, *B* and *C*).

## Discussion

A decade ago, AP2-G was identified as a master regulator of sexual commitment in two parasite species, namely *P. falciparum* (6) and the rodent parasite *P. berghei* (7). Since then, a series of bulk and single-cell transcriptomics studies have identified several putative effectors upstream and downstream of AP2-G, including early stages of gametocyte development. However, the environmentally sensitive decision to sexual commitment must be finely tuned and fast, hence transcriptional signatures may provide an incomplete picture of the process. To facilitate systematic analysis of sexually committed schizonts, we have successfully established a protocol for FACS-based isolation of sexually committed schizonts. For this purpose, we used an AP2-G reporter line and a novel reporter line expressing MSRP1 tagged with mNeonGreen. Here, we present the proteomic characterization of sexually committed schizonts compared to their non-committed counterparts using AP2-G and MSRP1 reporter lines combined with FACS separation of positive and negative populations. We have identified a shortlist of candidate proteins that are significantly enriched in either the sexually committed or the non-committed schizont population (Supplemental Table S3). Of particular interest are several enriched merozoite antigens, including MSRP1, MSP5, MSP11, DBLMSP and Rh2a.

MSRP1 is part of the MSP7 family that forms a monophyletic clade of 8 paralogs in *P. falciparum* and is encoded on one locus on chromosome 13 (44). The function of *P. falciparum* MSRP1 is currently unclear. However, an *msrp1* knock out line showed increased gametocyte production, suggesting a role for MSRP1 in sexual commitment (14). By contrast, deletion of *P. falciparum msp7* led to a significant reduction in merozoite invasion (45). Similarly, genetic deletion of the entire *msp7* locus in *P. berghei* (encompassing 3 paralogs) resulted in reduced parasite growth in the rodent model (45, 46). Here we demonstrate that MSRP1 is uniquely present in sexually committed schizonts and localized to the merozoite surface. Interestingly, one of the candidates identified by proteomics, MSP11 co-localized with MSRP1 on the merozoite surface of the sexually committed schizont. By contrast, it accumulated at the apical tip in non-committed schizonts. Both MSP11 localization patterns have previously been described (43, 47), and it was suggested that these represent early (internal staining) *versus* late (merozoite surface staining) asexual schizont stages. However, we demonstrate here that MSP11 protein levels are increased in sexually committed schizonts, where the protein predominantly localizes to the merozoite surface. In contrast, the majority of asexual (MSRP1 negative) schizonts shows internal punctate or diffuse staining, suggesting differences in its trafficking between the two parasite populations. Indeed, several protein trafficking factors show significantly increased abundance in sexually committed schizonts, including subtilisin 1 and 2, Rab5a, ARFGAP2 and SNF7. The function of MSP11 is currently unclear. While genetic deletion did not show any phenotype (47), functional assays using the polyclonal MSP11 antibodies suggested a role of MSP11 in merozoite invasion (43).

Another candidate from the proteomics data is Rh2a (its close paralog, Rh2b was just below the significance cutoff). Several studies support the role of Rh2a and Rh2b in alternative invasion pathways that mimic preferential invasion into reticulocytes v*s* normocytes (42, 48). Interestingly, Rh2a and Rh2b are encoded on chromosome 13 next to the *msp7* locus. Investigation of genetic determinants of alternative RBC invasion pathways by quantitative trait locus (QTL) mapping in progeny of genetic crosses identified the most significant LOD score in this locus (49). Moreover, the study also identified a minor peak on chromosome 10 in the *msp3* family locus that includes MSP11 and DBLMSP. Altogether these observations support a model where sexual merozoites are primed with putative invasion ligands (or a complex) that may facilitate an alternative RBC invasion pathway. While *msrp1* is activated by AP2-G and hence uniquely expressed in sexually committed schizonts, Rh2a/b and MSP11 are expressed in both sexually committed and non-committed schizonts. Interestingly, *msrp1* and *rh2a/b* are co-regulated by the transcription factor AP2-I that regulates expression of multiple invasion related genes (50), suggesting some interaction between the regulation of alternative invasion pathways and activation of sexual commitment. Both processes appear to respond to similar metabolic changes (11) and other external parameters such as static *vs* shaking culture conditions (51). Paralogs of the MSP3 and MSP7 families are under strong balancing selection, suggesting antibody-mediated immune responses and/or interaction with variable RBC receptors (52). Indeed, MSP11 antibody levels are higher in asymptomatic individuals compared to symptomatic patients in an area of low transmission in Thailand, hence anti MSP11 antibodies may play a role in protection against clinical malaria (43).

Apart from merozoite antigens and proteins involved in protein trafficking, additional candidates significantly enriched in sexually committed schizonts include cytoskeleton proteins required for merozoite invasion (myosin A, myosin E, myosin essential light chain, coronin) (53, 54, 55), two putative transporters (magnesium transporter NIPA and the Plasmodium surface anion channel [PSAC] (6, 22, 43) component CLAG8) and several proteins potentially involved in transcriptional regulation. In particular, a putative rRNA-processing protein (PF3D7_12254000) showed the greatest enrichment (>4-fold) of all hits.

A small set of proteins was significantly more abundant in non-committed schizonts. These include four out of the six components of the heterohexameric DNA prereplication complex MCM (MCM2-5) (56), a putative cell cycle associated protein and Clag 3.1. Like CLAG8, CLAG3.1 is part of the PSAC complex consisting of variable CLAG components and the two conserved proteins RhopH2 and RhopH3 (57, 58). PSAC is assembled at the RBC membrane post invasion and enables selective uptake of various solutes required for parasite metabolism, as well as antimalarial drugs and other compounds. To enter the parasite, nutrients and drugs that have entered the infected RBC through PSAC need to cross the parasitophorous vacuole membrane (PVM) and ultimately the parasite membrane (PM) (59). Apart from this role in transport and uptake, the smallest member of the PSAC channel, RhopH3 was also shown to play a role in invasion (60).

In summary, we present the first proteomic characterization of sexually committed vs non-committed schizonts in malaria parasites. The results suggest unique functional properties of the two populations, particularly the invasive daughter merozoite. Our study sets the stage for systematic comparative analysis of these parasite stages at a critical juncture in the parasite cycle.

## Data Availability

All relevant data have been deposited to the ProteomeXchange Consortium via the PRIDE (http://www.ebi.ac.uk/pride) partner repository with the data set identifier PXD059080 (AD1).

## Supplemental Data

This article contains supplemental data.

## Conflict of Interest

The authors declare that they have no conflicts of interests with the contents of this article.

## Supplementary Data

Supplemental Table S4

Proteomic_Checklist_V2
