# Supplementary material for: Defining the Proteome of Sexually Committed Parasites in Plasmodium falciparum
Source: Mol Cell Proteomics. 2025 Dec 31;25(2):101505. doi: 10.1016/j.mcpro.2025.101505 (PMC12878696; doi:10.1016/j.mcpro.2025.101505)
Supplement: Proteomic_Checklist_V2 [file mmc6.pdf]

MCP MOLECULAR & CELLULAR PROTEOMICS

All original manuscripts submitted to MCP that contain protein and post-translational modification identifications determined by mass spectrometry must be in compliance with our established guidelines for this type of data. Any required supplemental data may be submitted along with the manuscript. Public repositories that are not under the control of the authors or their collaborators are acceptable alternatives for raw data and annotated spectra. Manuscripts containing identification data will be checked to ensure they comply with the guidelines at the same time that the peer review is being performed. **The compliance check does not constitute a review of the manuscript as this ONLY determines if the article conforms to the guidelines, i.e., contains the requisite information.** The compliance check does not judge the quality of the data or evaluate the scientific suitability of the manuscript. Any compliance issues will be returned to the authors along with the scientific evaluations and must be addressed in any revised manuscript that is submitted.

To aid authors in preparing their manuscripts, the check list of the items that must be included is given below. Authors are encouraged to print out this document and use it to ascertain that the complete set of required information has been included. Authors submitting this type of data for the first time are particularly encouraged to use both the checklist and tutorial. For more information on how to provide access to annotated spectra, click here.

Check list for Publication of Peptide & Protein Identification Data in MCP:

The following information must be included in the *Experimental Procedures* section:

**Experimental Design and Statistical Rationale:**			**Page**
Authors provided a subsection in the *Experimental Procedures* section with the header "*Experimental Design and Statistical Rationale*" that contains the following information:	Y	N/A	9
The sample size (n) (for each experiment), as a number (ranges are not acceptable)	Y	N/A	9
The rationale for that (n) choice (statistical power of detection or similar)	Y	N/A	9
Numbers and types of controls employed	Y	N/A	9
The number of replicates acquired, including a clear distinction between biological (independent data points), process and technical replicates, and rationale for that choice	Y	N/A	9
The criteria for the inclusion or exclusion of data points (if relevant)	Y	N/A	9
A description of the statistical methods used for analysis	Y	N/A	9
Justification for the statistical methods used for analysis (e.g. has the dataset a normal distribution? etc.)	Y	N/A	9

**Search Parameters and Acceptance Criteria (MS/MS and/or Peptide Mass Fingerprint (PMF) data):**			**Page**
Name of peaklist-generating software and release version (number or date)	Y	N/A	15
Name of the search engine and release version (number or date)	Y	N/A	15

Name of sequence database/spectral library searched and release version/date	Y	N/A	15
If the database was generated in-house, source of sequences and software used to compile it	Y	N/A	NA
# of entries in the database (or subset of database) actually searched	Y	N/A	15
Specificity of all proteases used to generate peptides	Y	N/A	15
# of missed and/or non-specific cleavages permitted	Y	N/A	15
List of all fixed modification(s) (including residue specificity) considered	Y	N/A	15
List of all variable modification(s) (including residue specificity) considered	Y	N/A	15
Mass tolerance for precursor ions	Y	N/A	15
Mass tolerance for fragment ions (not required for PMF data)	Y	N/A	15
Known contaminants excluded (primarily for PMF data)	Y	N/A	
Threshold score/Expectation value for accepting <u>individual</u> spectra	Y	N/A	15
Justification of the threshold score/expectation value employed	Y	N/A	NA
Estimation of false discovery rate (FDR) (for large datasets) and how calculated	Y	N/A	NA

The following information must be included in the *Results* or *Supplemental* sections:

**Peptide and Protein Identification**			**Page**
For all peptide sequences assigned:			
List (in tabular form) of all peptide sequences, including any deviations from expected cleavage specificity	Y	N/A	Table S3
Precursor charge and mass/charge (m/z) for each assignment	Y	N/A	NA
All modifications observed	Y	N/A	NA
# of matched and unmatched masses (for PMF data)	Y	N/A	NA
Peptide Identification Score(s)	Y	N/A	NA
For all protein identifications:			
Accession # (and database from which it is derived)	Y	N/A	Table S3

# of distinct peptides assigned for each protein	Y	N/A	Table S3
% coverage of each protein assigned (or derived protein identification probability)	Y	N/A	NA
Single peptide and PMF identifications, annotated spectra are provided for each protein:	Y	N/A	NA
In a publicly accessible database (and database reference/entry number is provided) (For more info: Providing Access to Annotated Spectra)	Y	N/A	NA
Submitted as supplemental material with the manuscript	Y	N/A	NA

**Post-Translational Modifications**			**Page**
Site(s) of modification within each peptide clearly located	Y	N/A	NA
Justification of any localization score threshold	Y	N/A	NA
Separate table for all peptides with ambiguous modification site assignments	Y	N/A	NA
Annotated, mass labeled spectra for all modified peptides are provided:	Y	N/A	NA
In a publicly accessible database (and database reference/entry number is provided) (For more info: Providing Access to Annotated Spectra)	Y	N/A	NA
Submitted as supplemental material with the manuscript	Y	N/A	NA

**Protein Inference from Peptide Assignments**			**Page**
Accession numbers provided for all proteins that were combined into a group	Y	N/A	
Indication of which peptides are distinct and which are shared within a group of proteins	Y	N/A	
Indication of all proteins identified from different species than the one under investigation (based on homology)	Y	N/A	

**Quantification**			**Page**
Quantification measurements for each peptide and protein	Y	N/A	Table S3
Description of how raw mass spectrometric data was processed to yield quantification data	Y	N/A	15
Analytical reliability described (technical replicates and statistical treatments)	Y	N/A	15
Biological reliability described (biological replicates, independent experiments, statistical analyses)	Y	N/A	9
Description of any adjustments for systematic errors	Y	N/A	15

How random error issues were addressed (outliers, exclusion limits etc.)	Y	N/A	NA
Estimates of uncertainty for individual proteins	Y	N/A	15
How the identity of the analyte was verified (in non-database identifications)	Y	N/A	NA
How quantification of multiple isoforms in the same sample was handled	Y	N/A	NA

For spectral counting:

# of peptides or # of spectra used for the quantification	Y	N/A	NA
Inclusion of modified, semi-tryptic or shared peptides (from different isoforms)	Y	N/A	NA

Raw Data Submission

The raw mass spectrometric data has been deposited **Y**

The location and identifying information (URL of repository, deposit ID, username, hash code/identifier, password) are:

Note: The reviewers' login information (username, password) to access the raw data also needs to be included in the cover letter
